# HHV-6B Induces IFN-Lambda1 Responses in Cord Plasmacytoid Dendritic Cells through TLR9

**DOI:** 10.1371/journal.pone.0038683

**Published:** 2012-06-06

**Authors:** Inger Nordström, Kristina Eriksson

**Affiliations:** Department of Rheumatology and Inflammation Research, Sahlgrenska Academy, University of Gothenburg, Gothenburg, Sweden; Karolinska Institutet, Sweden

## Abstract

Human herpesvirus type 6B (HHV-6B) is a strong inducer of IFN-alpha and has the capacity to promote Th1 responses and block Th2 responses *in vitro*. In this study we addressed whether inactivated HHV-6B can also induce IFN lambda responses and to what extent interferons alpha and lambda affect Th1/Th2 polarization. We show that inactivated HHV-6B induced IFN-lambda1 (IL-29) but not IFN-lambda2 (IL-28A) responses in plasmacytoid DC and that this induction was mediated through TLR9. We have previously shown that HHV-6B promotes Th1 responses and blocks Th2 responses in both humans and mice. We now show that neutralization of IFN-alpha but not IFN-lambda1 blocked the HHV-6B-induced enhancement of Th1 responses in MLR, but did not affect the HHV-6-induced dampening of Th2 responses. Similarly, blockage of TLR9 counteracted HHV-6Bs effects on the Th1/Th2 balance. In addition, IFN-alpha but not IFN-lambda1 promoted IFN-gamma production and blocked IL-5 and IL-13 production in purified CD4+ T-cells. The lack of effect of IFN-lambda1 correlated with the absence of the IFN-lambda receptor IL-28Ralfa chain on the cell surface of both resting and activated CD4+ T-cells. We conclude that inactivated HHV-6B is a strong inducer of IFN-lambda1 in plasmacytoid DC and that this induction is TLR9-dependent. However, human CD4+ T-cells do not express the IFN-lambda receptor and are refractory to IFN-lambda1 treatment. The HHV-6B-induced alterations in the Th1/Th2 balance are instead mediated mainly through TLR9 and IFN-alpha.

## Introduction

The recently discovered type III IFNs share many of the anti-viral properties of type I IFNs. The family consists of three members, IFN-lambda1 (IL-29), IFN-lambda2 (IL-28A), and IFN-lambda3 (IL-28B) and are commonly referred to as the IFN-lambda family [1], [2]. All three IFN-lambdas utilize a common distinct receptor, the IFN-lambdaR, which is composed of the IL-10R2 chain and the unique IL-28Ralpha chain [2], [3]. The IFN-lambdaR is expressed at high densities on epithelial cells [3] but IL-28Ralpha transcripts might also be found in other cells including blood lymphocytes [2]. Binding of IFN-lambda to its receptor induces a signaling response similar to that obtained by type I IFNs [1], even though the intensity of the IFN-lambda response is generally weaker [1], [4]. The anti-viral effects obtained by IFN-lambda protects against some but not all viral infections, and preferentially those that occur at mucosal surfaces [5], [6].

Type I IFNs are pleiotropic and have, in addition to their anti-viral properties, also T-cell immune-modulatory functions, e.g. in driving Th1 responses. IFN-alpha not only promotes IFN-gamma production by CD4+ and CD8+ T-cells [7], [8] but can also re-direct an already established Th2 response into a more Th1-like response [9]. IFN-lambda share some of the immune-modulatory functions of IFN-alpha. Signaling through the IFN-lambdaR induces the phosphorylation of both STAT 1 and STAT 4 [1], [4], i.e. transcription factors involved in the development of IFN-gamma/Th1 responses [7], [10]. The main T-cell immune-modulatory function of IFN-lambda1 that has been reported is in dampening Th2 responses, in particular IL-13 [11]–[13]. It is however not conclusively proven that IFN-lambda has direct effects on CD4+ T-cell polarization rather than operating through accessory cells such as dendritic cells and monocytes [14].

Human herpesvirus type 6B (HHV-6B) is the aetiological cause of *exanthema subitum*, a benign disease in young children characterized by high fever for 3–5 days. The virus is a strong inducer of type I IFN responses [15] and exposure to this virus alters the Th1/Th2 balance both *in vitro* and *in vivo* [15], [16]. Whether HHV-6B is also capable of inducing IFN-lambda responses is not known, and neither is the underlying mechanism for the Th1-promoting and Th2-dampening effects on CD4+ T-cell responses.

In this study we investigated whether inactivated HHV-6B could induce the production of IFN-lambda by cord plasmacytoid dendritic cells (pDC), and to what extent this production contributed to the documented shift in the Th1/Th2 balance. We show that inactivated HHV-6B induces the production of IFN-lambda1 by cord pDC in a TLR9-dependent fashion. IFN-lambda however had no direct effect on human cord T-cell polarization, which correlates with the lack of expression of the IL-28Ralpha on human CD4+ T-cells. We conclude that IFN-lambda1 has no direct effects on the Th1/Th2 differentiation of human CD4+ T-cells.

## Results

### Inactivated HHV-6B induces IFN-alpha and IFN-lambda1 responses in cord pDC via TLR9

**Figure 1:**
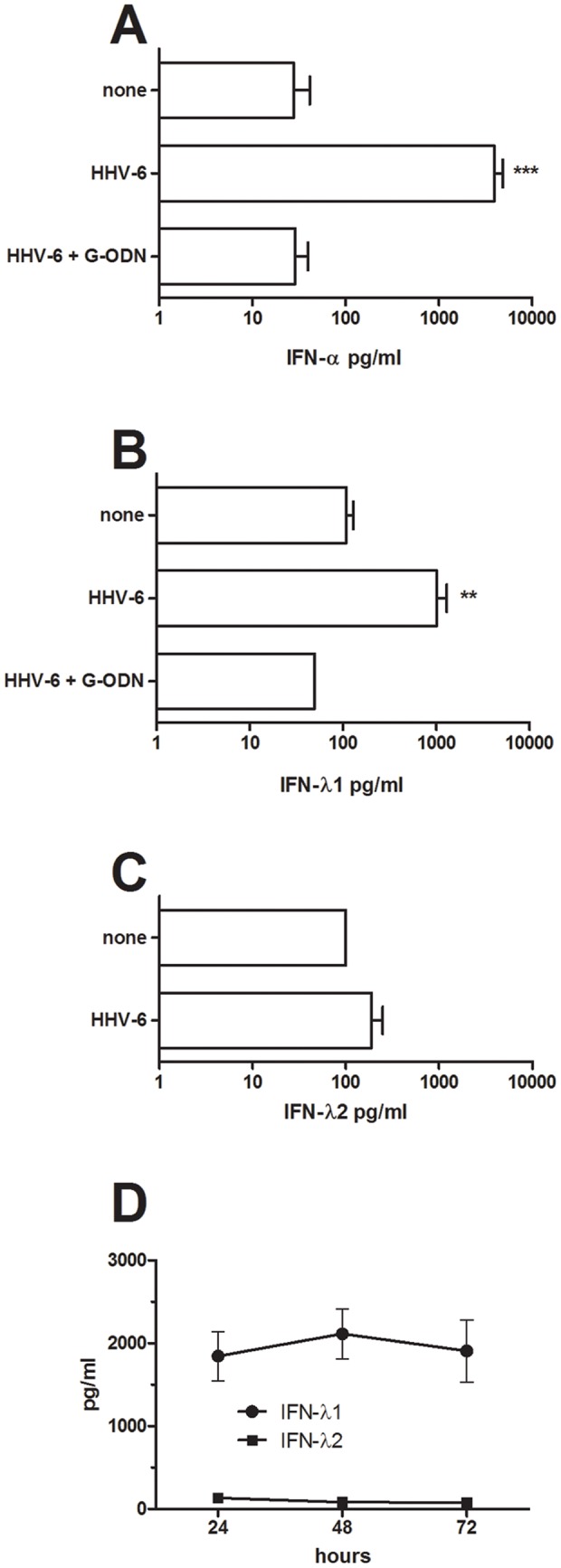
HHV-6B induces the production of IFN-alpha, IFN-lambda1 but not IFN-lambda2 in pDC in a TLR9-dependent fashion. Purified cord pDC were exposed to inactivated HHV-6B in the presence or absence of G-ODN, a TLR9-specific inhibitor. The levels of IFN-alpha (A; n = 20), IFN-lambda1 (B; n = 15) and IFN-lambda2 (C; n = 5) were analyzed in 24h culture supernatants. Data are expressed as mean + SEM. **  =  p<0.01, ***  =  p<0.001 using ANOVA with Bonferroni's multiple comparison test. The levels of IFN-lambda1 and IFN-lambda2 were also assessed over a 72 h time-period (D; n = 3).

We have previously shown that inactivated HHV-6B induces a strong IFN-alpha response in cord pDC, which we confirm in Fig. 1A. In addition, we show that inactivated HHV-6B also induces high levels of IFN-lambda1 from pDC (Fig. 1B), but no or little IFN-lambda2 (Fig. 1C). The lack of induction of IFN-lambda2 was not dependent on the kinetics, at these low levels remained constant over 72 h (Fig. 1D). It should be noted that the sensitivity of the ELISAs varied from 10 pg/ml for IFN-alpha, 60 pg/ml for IFN-lambda1 and 100 pg/ml for IFN-lambda2. This means that we have not been able to detect potential low IFN-lambda2 responses. The HHV-6B-induced production of both IFN-alpha and IFN-lambda1 was dependent on TLR9 as it was completely abolished in the presence of a TLR9-specific inhibitor, G-ODN (Fig. 1A–B).

### Neutralization of IFN-alpha but not IFN-lambda1 blocks the HHV-6B-induced promotion of Th1 responses in cord mixed lymphocyte reactions without affecting the HHV-6-induced dampening of Th2 responses

**Figure 2:**
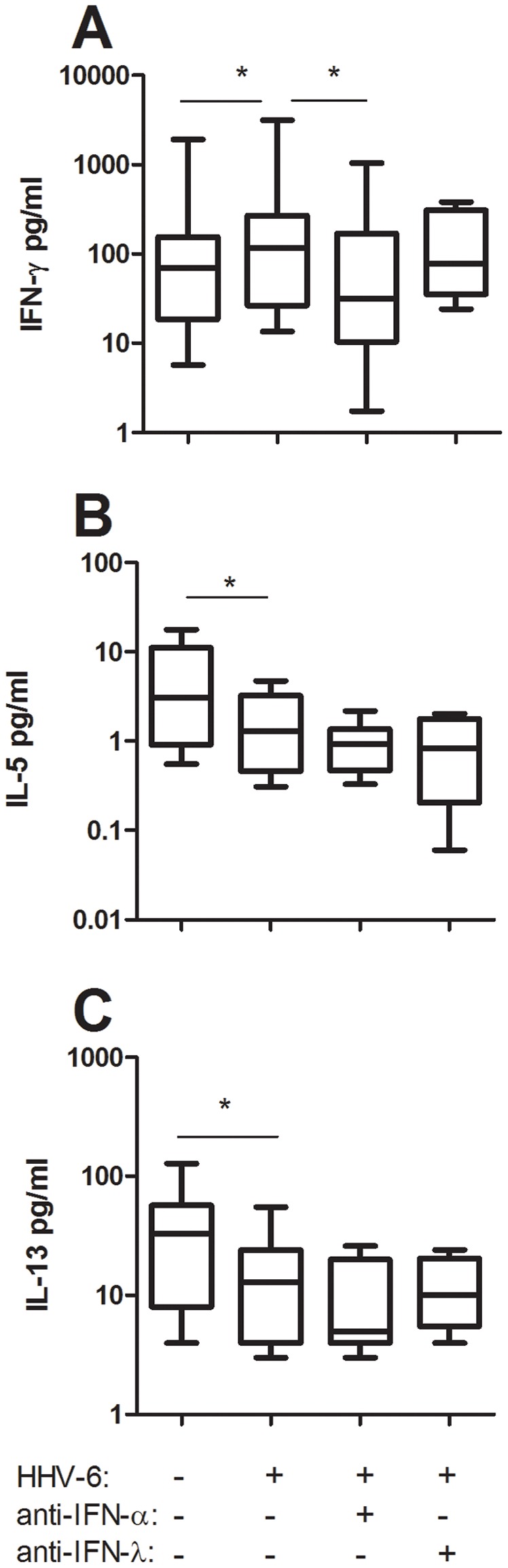
Neutralization of IFN-alpha but not IFN-lambda1 blocks the HHV-6B-induced promotion of IFN-gamma responses in cord mixed lymphocyte reactions. Cord blood pDC were incubated with allogeneic cord-blood CD4+ T-cells in the presence or absence of inactivated HHV-6B and neutralizing antibodies to IFN-alpha or IFN-lambda1. Supernatants were collected after 48 h and analyzed for IFN-gamma (A), IL-5 (B) and IL-13 (C) content. Data are expressed as medians and the 25% and 75% percentile (the boxes) with the minimum and maximum responses for n = 9. *  =  p<0.05 using ANOVA with Bonferronís multiple comparison test.

**Figure 3:**
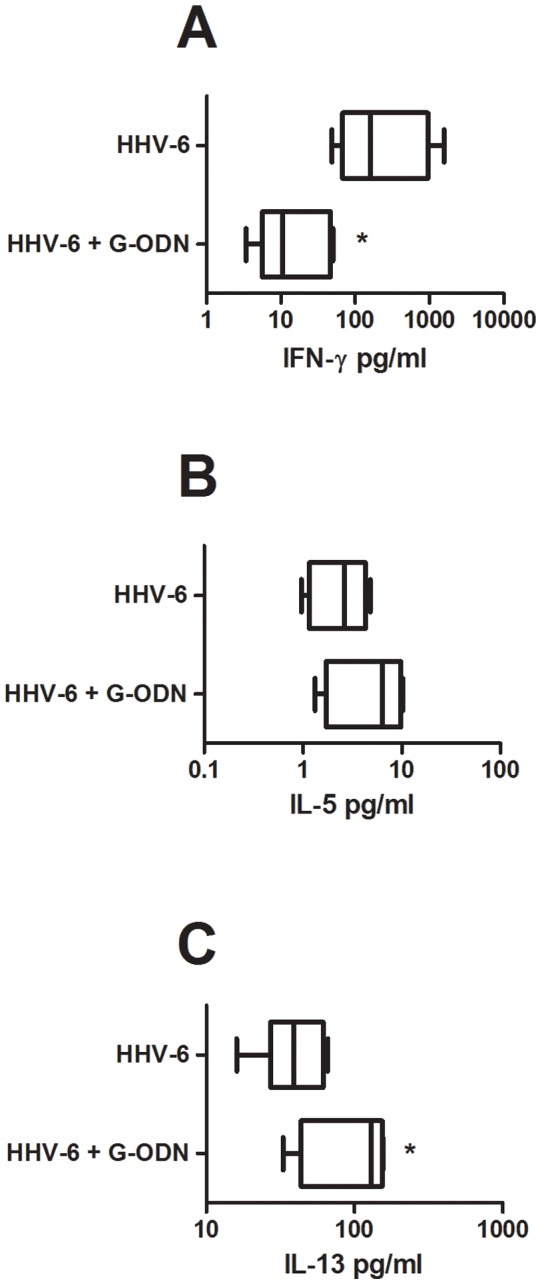
HHV-6B affects the IFN-gamma and IL-13 responses in cord mixed lymphocyte reactions via TLR9 signaling. Cord blood pDC were incubated with allogeneic cord-blood CD4+ T-cells and inactivated HHV-6B in the presence or absence of G-ODN. Supernatants were collected after 48 h and analyzed for IFN-gamma (A), IL-5 (B) and IL-13 (C) content. Data are expressed as medians and the 25% and 75% percentile (the boxes) with the minimum and maximum responses for n = 5. *  =  p<0.05 using student's t-test.

We have previously shown that HHV-6B exposure promotes Th1 development and blocks Th2 development both *in vitro* and *in vivo* in humans and in mice [15], [16]. To assess whether IFN-alpha or IFN-lambda1 is implicated in the HHV-6B-induced Th1/Th2 shift *in vitro*, we performed MLR of cord pDC and CD4+ T-cells in the presence or absence of HHV-6B and either a specific inhibitor of TLR9 (G-ODN) or neutralizing antibodies to IFN-alpha or IFN-lambda1. In titration experiments, we found that at 10 ug/ml, both these antibodies neutralized 5000 pg/ml of recombinant IFN-alpha and IFN-lambda1, respectively (not shown). As shown before [15], the CD4+ T-cell production of IFN-gamma was increased while the production of IL-5 and IL-13 was decreased in MLR cultures exposed to HHV-6B (Fig. 2A–C). Neutralizing antibodies to IFN-alpha blocked the HHV-6B-induced IFN-gamma responses while neutralizing antibodies to IFN-lambda1 did not have this effect (Fig. 2A). Addition of G-ODN, a specific blocker of TLR9 signaling, blocked the HHV-6B-induced increase in IFN-gamma production and also the HHV-6-induced dampening of IL-13 responses in these cultures (Fig. 3). Neutralization of IFN-alpha or IFN-lambda1 did however not impact on the HHV-6B-induced dampening of IL-5 and IL-13 responses (Fig. 2B–C).

### IFN-alpha but not IFN-lambda1 promotes IFN-gamma responses and blocks IL-5 and IL-13 responses in activated cord CD4+ T-cells

**Figure 4:**
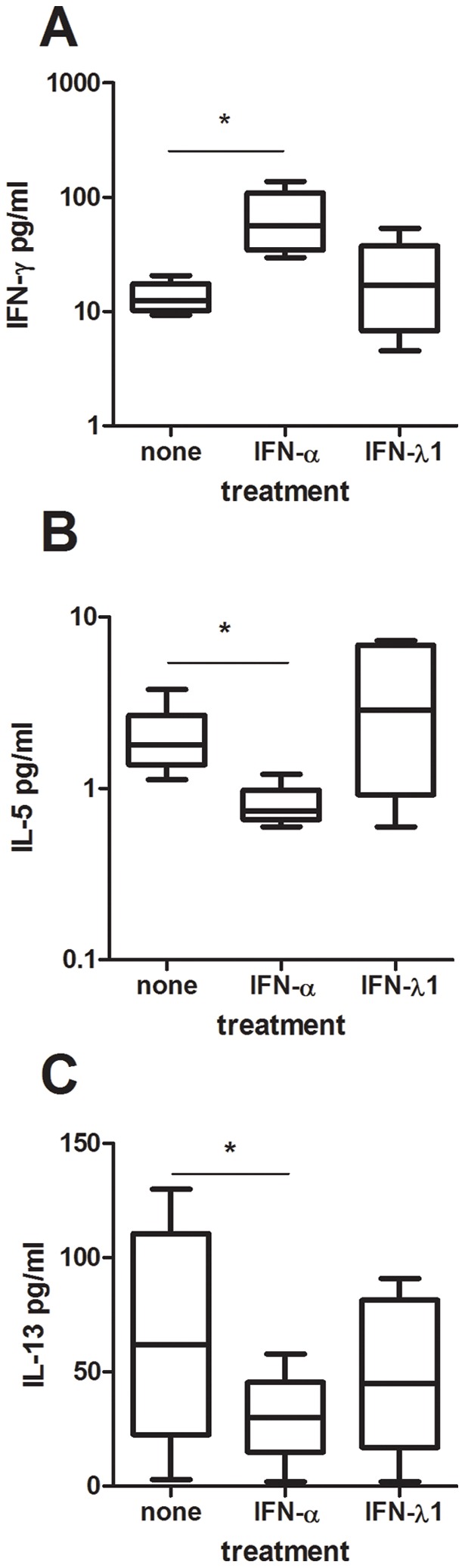
IFN-alpha but not IFN-lambda1 promote Th1 responses and block Th2 responses in activated cord CD4+ T-cells. Cord blood CD4+ T-cells were activated with anti-CD3 (A–B) or anti-CD3 and anti-CD28 (C) in the presence or absence of recombinant human IFN-alpha or IFN-lambda1. Supernatants were collected after 48 h and analyzed for IFN-gamma (A), IL-5 (B) and IL-13 (C) content. Data are expressed as medians and the 25% and 75% percentile (the boxes) with the minimum and maximum responses for n = 5. *  =  p<0.05 using ANOVA with Bonferroni's multiple comparison test.

To assess whether IFN-lambda1 shares the CD4+ T-cell immune-modulatory functions of IFN-alpha, we added recombinant human IFN-alpha and IFN-lambda1 to cultures of anti-CD3-stimulated cord CD4+ T-cells and measured their production of IFN-gamma, IL-5 and IL-13. To ascertain that the recombinant cytokines used were biologically functional we first assessed their ability to induce MxA transcription in purified pDC and found that four hour incubation with IFN-alpha or IFN-lambda1 enhanced the MxA transcription 50-fold and 10-fold, respectively. Since anti-CD3 activation alone did not give rise to any measurable IL-13 responses (not shown), we added soluble anti-CD28 to these cultures. As expected, IFN-alpha had profound effects on the cytokine production profiles and promoted both IFN-gamma and IL-10 responses and blocked IL-5 and IL-13 responses by the T-cells (Fig. 4). IFN-lambda1 on the other hand did not affect the production of any of these cytokines (Fig. 4).

### IL28R is not expressed on CD4+ T-cells

**Figure 5:**
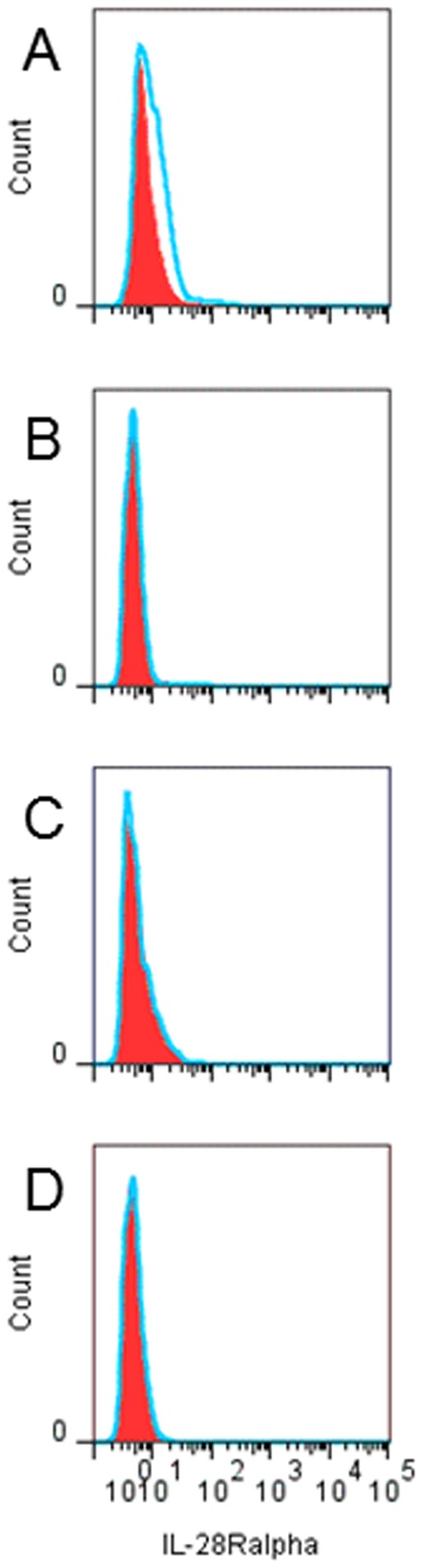
CD4+ T-cells do not express IL-28Ralpha. Resting or activated cord blood mononuclear cells were analyzed for IL-28Ralpha expression using FACS. IL-28Ralpha expression (histograms) on pDC (A) or CD4+ T-cells obtained directly upon isolation (B) or after 24 h incubation with anti-CD3 (C) or recombinant human IFN-alpha (D). Filled red areas represent isotype-PE control and blue lines represent IL-28Ralpha-PE.

IFN-lambda1, IFN-lambda2 and IFN-lambda3 act by binding to the IFN-lambdaR. We examined the expression of the IFN-lambdaR-specific IL-28Ralpha chain on pDC (Fig. 5A), resting CD4+ cells (Fig. 5B) and on CD4+ cells that had been activated for 24 h with anti-CD3 (Fig. 5C) or cultured for 24 h in the presence of IFN-alpha (Fig. 5D). We found that neither resting nor activated CD4+ T-cells expressed any detectable levels of IL-28Ralpha whereas low levels could be detected on pDC.

## Discussion

In this study we show that inactivated HHV-6B is a potent inducer of IFN-lambda1 but not IFN-lambda2 in pDC through a TLR9-dependent signaling pathway. In addition we show that IFN-lambda1 does not share the ability if IFN-alpha to directly alter the function of T-cells. Firstly, T-cells do not express IL-28Ralpha, i.e. the IFN-lambdaR. Secondly, addition of recombinant IFN-lambda to activated CD4+ T-cells does not change their Th1/Th2 cytokine profile. Thirdly, neutralization of IFN-lambda1 does not affect virus-induced alterations in the Th1/Th2 balance.

Inactivated HHV-6B was a strong inducer of IFN-lambda1 but not IFN-lambda2 in cord pDC. Even though IFN-lambda1 genes are considered to be early response genes whereas IFN-lambda2 is expressed with delayed kinetics [14], there were no apparent IFN-lambda2 responses even at 72 h post activation. IFN-lambda2 depends on the inducible IRF7 pathway whereas IFN-lambda1 is also regulated by the constitutively expressed IRF3 [17]. Since IRF7 is constitutively expressed in pDC [18], our data clearly show that pDC produce higher amounts of IFN-lambda1 compared to IFN-lambda2 in response to HHV-6B exposure. We have previously shown that inactivated HHV-6B can induce high levels of IFN-alpha by cord pDC [15] and the data from this study show that both the IFN-alpha and the IFN-lambda1 responses induced by HHV-6B are mediated through TLR9. This is in accordance with many other herpesviruses such as varicella zoster virus, HSV and EBV, which all induce type I IFN responses in pDC through the TLR9 pathway [19]–[21].

We confirm previous studies showing that IFN-alpha has the capacity to alter the overall Th1/Th2 balance. That IFN-alpha has T-cell immune-modulatory functions is well established [7]–[9] and reflects the almost universal expression of the IFN-alpha/beta receptor on nucleated cells. However, when we neutralized IFN-alpha in HHV-6B-exposed mixed lymphocyte reactions we only managed to influence the Th1 responses. Thus, addition of neutralizing antibodies to IFN-alpha blocked the virus-induced enhancement in IFN-gamma production without affecting the virus-induced reduction in Th2 cytokines. In contrast, addition of recombinant IFN-alpha to activated cord CD4+ T-cells affected both the Th1 and the Th2 responses. In this setting, IFN-alpha enhanced the production of IFN-gamma and reduced the production of IL-5 and IL-13. This implies that IFN-alpha indeed has the capacity to influence both Th1 and Th2 responses. The lack-of-effect of anti-IFN-alpha in enhancing the Th2 responses in the HHV-6B-exposed cord MLR could imply that other factors than IFN-alpha also has a Th2-dampening effect. In relation to this, we recently showed that enveloped viruses (in this case coronavirus, CMV, HSV-1, influenza virus and morbillivirus), but not naked virus, blocked IL-13 responses *in vitro*, and that this inhibition occurred independently of IFN-alpha responses [22]. This would argue for an IFN-alpha-independent modulation of IL-13 responses by virus such as HHV-6B.

IFN-lambda1 is reported to be especially efficient in dampening Th2 responses, in particular IL-13 [11]–[13]. In our hands, IFN-lambda1 had no such effect. When we added recombinant IFN-lambda1 to activated cord CD4+ T-cells, we could not document any effect on either Th1 (IFN-gamma) or Th2 (IL-5 and IL-13) responses. In addition, neutralization of IFN-lambda1 in HHV-6B-exposed cord mixed lymphocyte reactions did not affect the levels of either Th1 or Th2 cytokines, including IL-13. We therefore decided to check for the expression of the IFN-lambda receptor on CD4+ T-cells. Resting CD4+ T-cells did not show any expression of the IL-28Ralpha chain, nor did CD4+ T-cells that had been activated with anti-CD3 or exposed to IFN-alpha. Previous studies have identified IL-28Ralpha transcripts in human T-cells [13], [23] but our data clearly show that there is no IL-28Ralpha protein expression on the cell surface on neither resting nor activated CD4+ cells. We thus conclude that IFN-lambda1 does not have direct effects on CD4+ T-cells due to the lack-of-expression of the cognate cytokine receptor. This is in line with a recent report by Arasteh et al [24] showing that IFN-lambda2 has no effect on CD25 expression or proliferation of cord CD4+ T-cells. There is however one report showing a direct effect of IFN-lambda1 on purified human CD4+ T-cells [13]. In this study it was shown that IFN-lambda not only suppressed GATA-3-induced Th2 responses but also indirectly enhanced IFN-gamma responses [13]. However, the authors used negative selection for the CD4+ purification. Given that the purified cells used were not completely pure (>95%) indicates that there might have been some accessory cells left among the CD4+ T-cells [13]. If so, the effect of IFN-lambda1 could well be mediated indirectly via some other cell subset.

Monocyte-derived macrophages not monocytes or monocyte-derived DC express the functional IFN-lambdaR [25]. The transcript for the IL-28Ralpha has also been found in pDC [26] and we now show that pDC express low levels of IL28Ralpha on the cell surface. This indicates to us that IFN-lambda acts preferentially on certain subsets of antigen-presenting cells and that the observed effects of IFN-lambda on CD4+ T-cell differentiation [11]–[13] are indirect. The most plausible mode of action of IFN-lambda is through its interaction with myeloid cells [27]. This assumption is substantiated by *in vitro* studies showing that IFN-lambda blocks IL-13 responses and promotes IFN-gamma responses through its effects on dendritic cells [11], [12]. Furthermore, IFN-lambda promotes Th1 responses and blocks Th2 and Th17 responses *in vivo* in mice through its modulation of the IL-12 production by lung myeloid dendritic cells [28]. The Th2 dampening and Th1 promoting effects could be adoptively transferred by IL-28A-treated CD11c^+^ cells [28].

We have recently shown that infection with HHV-6B before 18 months-of-age is associated with a significantly reduced incidence of allergic sensitization (OR: 0.08, 95% CI: 0.009–0.68) [15] suggesting that HHV-6B infection might protect children from allergy. Experimentally, we strengthened this hypothesis by showing that HHV-6B exposure blocks Th2 responses and allergy development in a mouse model of allergic disease [16]. *In vitro* we showed that HHV-6B can promote Th1 responses (IFN-gamma) and reduce Th2 responses (IL-5 and IL-13) [15]. The results from the present study indicate that these effects are mediated in part through IFN-alpha. We initially hypothesized that IFN-lambda is implicated in dampening the Th2 responses [11]–[13], but this was not the case in our *in vitro* system. However, IFN-lambda might well play a role in reducing Th2 responses *in vivo*, as shown by Koltsida et al [28].

In summary, we show that inactivated HHV-6B induces IFN-lambda1 responses in pDC through TLR9. The secreted IFN-lambda1 does however not affect CD4+ T-cells directly as human CD4+ T-cells do not express the IFN-lambdaR and are refractory to IFN-lambda1 treatment.

## Materials and Methods

### Virus

HHV-6B strain Z29 (available from the HHV-6 foundation repository, www.hhv-6foundation.org/research/repository/repository-reagents-available) was kindly donated by Dr Helena Dahl, SMI, Stockholm. The virus was grown in MOLT-3 cells (available at ATCC: CRL 1552) in Iscoves complete medium (Iscoves medium with 10% FBS (Biological Industries, Kibbutz Beit Haemek, Israel), 3 ug ml^−1^ L-glutamine (Gibco, UK), 0.1 mg ml^−1^ gentamycin sulphate (Essex Läkemedel AB, Stockholm, Sweden)) containing 5 ug/ml cortisone (Solucortef, UpJohn). After 2–3 weeks, the suspensions were centrifuged at 1500 rpm. The virus-containing supernatants were then centrifuged for 1 h at 100,000×*g*. The viruses were resuspended in Iscoves complete medium. Viral content was determined by quantitative PCR. The virus was inactivated at 2500 rad prior to use.

### Activation of pDC

Fresh cord blood pDC were isolated from cord blood mononuclear cells using the pDC isolation kit from Miltenyi Biotec (Auburn, CA). PDC were dispersed in 24 well plates (10^6^ cells/well) in the presence or absence of virus (70 genome copies/pDC) in 1 ml Iscoves complete medium. In some experiments, a TLR9 antagonist (G-ODN, 15 uM, InVitrogen) [29] was added. Supernatants were collected after 24 h, 48 h and 72 h and frozen at −70°C until assayed for cytokine content.

### Activation of CD4+ cells

Cord CD4+ T-cells were isolated from pDC-depleted CBMC using the Dynal CD4^+^ Isolation Kit according to manufacturer's instructions (Invitrogen AB, Stockholm, Sweden) yielding >95% pure CD4+ T-cells. The cells were cultured in 96 well flat-bottom Nunc tissue culture plates (10^5^ cells/well) pre-coated with 3 ug/ml anti-human CD3 (3 ug/ml, OKT-3, Ortho-McNeil Pharmaceutical, Raritan, NJ) in Iscoves complete medium in the presence or absence of anti-human CD28 (1 ug/ml, BD), recombinant human IFN-alpha (10 ug/ml; PBL interferon source) or IFN-lambda1 (10 ug/ml; Peprotech). Supernatants were collected after 48h and analyzed for cytokine content (see below).

### Mixed lymphocyte reaction

Cord CD4+ T-cells (10^5^) were cultured with allogeneic cord pDC (2×10^4^) in 96 well flat-bottom Nunc tissue culture plates in Iscoves complete medium together with irradiated HHV-6 (70 genome copies/pDC), in the presence or absence of G-ODN (15 uM) or neutralizing antibodies to human IFN-alpha (10 ug/ml; PBL interferon source) or IFN-lambda1 (10 ug/ml; R&D, catnr: MAB15981). Supernatants were collected after 48 h and analyzed for cytokine content (see below).

### Cytokine analysis

IFN-alpha content was assessed using an ELISA kit from PBL Biomedical Laboratories. IFN-lambda1, IFN-lambda2, and IL-13 levels were determined using specific Duoset ELISAs (R&D). IFN-gamma and IL-5 contents were determined using the Human Th1/Th2 Cytokine Cytometric Bead Array Kit (BD Biosciences Pharmingen, San Diego, CA) using a FacsCanto2 flow cytometer and the data were analysed using the FCAP array Software (BD Biosciences Pharmingen).

### FACS analysis

Cord cells were analyzed directly upon isolation or after 24 h incubation with IFN-alpha (5 ug/ml) or solid-phase anti-CD3 (3 ug/ml). For the staining, 10^6^ cells were treated for 20 min with Fc-block (BD Pharmingen) and then incubated with anti-IL28R-PE (R&D, catalogue number FAB5260P) or a PE-labelled isotype control (mouse IgG) together with anti-CD4-Pacific blue (BioLegend) for 30 min. Stained cells were analyzed in an iCyt Eclipse (Sony) and the data generated were processed with FlowJo version 7.6.5.

### Relative quantification of MxA mRNA

Total RNA was isolated from pDC that had been cultured for 6 h with recombinant IFN-alpha or IFN-lambda1, using the RNeasy kit from Qiagen according to manufacturer's instructions. Total RNA was used for cDNA synthesis using 0.5 µg/ml random hexamer and 0.01 mM dNTP in a total volume of 12 µl. This was followed by heating to 65°C for 5 minutes, a quick chill on ice and brief centrifugation. 20–40 U/µl RNase inhibitor, 0.2 mM DTT, 1x First-Strand buffer and 200 U/µl SuperscriptRT was then added and incubated at 37°C for 50 minutes, followed by termination of the reaction at 70°C, 15 min. MxA mRNA expression levels was determined using a TaqMan® Gene Expression Assay (Applied Biosystems). Primers and probes for MxA and the housekeeping gene GAPDH were purchased from Applied Biosystems, cat no. Hs00895608_m1 and Hs99999905_m1, respectively. Each qPCR was carried out in a total volume of 20 µl containing 10 ng cDNA, 0.9 µM of each primer and 0.25 µM probe under the following conditions: 50°C for 2 min, 95°C for 10 min, 95°C for 15 s, 60°C for 1 min in 40 cycles. Data was analyzed using the 7500 system software for the 7500 real time PCR system and is presented as the ratio of MxA amplificied transcripts to GAPDH amplified transcripts.

### Statistical evaluations

Student's t-test and ANOVA with Bonferronís multiple comparison test were performed using GraphPad Prism version 5 for Window (GraphPad Software, San Diego, USA).

### Ethical approval

These studies were approved by The Ethics Committee at the Faculty of Medicine, University of Gothenburg, Sweden. All mothers were given oral and written information, and gave oral consent to participate in the study. Written consent was not required by the Ethics Committee for this study as no personal information or identity was recorded, and the cord blood was collected from the umbilical cord after the birth of the child (law 2003: 460, paragraphs 4 and 13).

## References

[1] Kotenko SV, Gallagher G, Baurin VV, Lewis-Antes A, Shen M (2003). IFN-lambdas mediate antiviral protection through a distinct class II cytokine receptor complex.. Nat Immunol.

[2] Sheppard P, Kindsvogel W, Xu W, Henderson K, Schlutsmeyer S (2003). IL-28, IL-29 and their class II cytokine receptor IL-28R.. Nat Immunol.

[3] Sommereyns C, Paul S, Staeheli P, Michiels T (2008). IFN-lambda (IFN-lambda) is expressed in a tissue-dependent fashion and primarily acts on epithelial cells in vivo.. PLoS Pathog.

[4] Dumoutier L, Tounsi A, Michiels T, Sommereyns C, Kotenko SV (2004). Role of the interleukin (IL)-28 receptor tyrosine residues for antiviral and antiproliferative activity of IL-29/interferon-lambda 1: similarities with type I interferon signaling.. J Biol Chem.

[5] Ank N, West H, Bartholdy C, Eriksson K, Thomsen AR (2006). Lambda interferon (IFN-lambda), a type III IFN, is induced by viruses and IFNs and displays potent antiviral activity against select virus infections in vivo.. J Virol.

[6] Mordstein M, Kochs G, Dumoutier L, Renauld JC, Paludan SR (2008). Interferon-lambda contributes to innate immunity of mice against influenza A virus but not against hepatotropic viruses.. PLoS Pathog.

[7] Nguyen KB, Watford WT, Salomon R, Hofmann SR, Pien GC (2002). Critical role for STAT4 activation by type 1 interferons in the interferon-gamma response to viral infection.. Science.

[8] Pien GC, Nguyen KB, Malmgaard L, Satoskar AR, Biron CA (2002). A unique mechanism for innate cytokine promotion of T cell responses to viral infections.. J Immunol.

[9] Farkas L, Kvale EO, Johansen FE, Jahnsen FL, Lund-Johansen F (2004). Plasmacytoid dendritic cells activate allergen-specific TH2 memory cells: modulation by CpG oligodeoxynucleotides.. J Allergy Clin Immunol.

[10] Xu X, Sun YL, Hoey T (1996). Cooperative DNA binding and sequence-selective recognition conferred by the STAT amino-terminal domain.. Science.

[11] Jordan WJ, Eskdale J, Srinivas S, Pekarek V, Kelner D (2007). Human interferon lambda-1 (IFN-lambda1/IL-29) modulates the Th1/Th2 response.. Genes Immun.

[12] Srinivas S, Dai J, Eskdale J, Gallagher GE, Megjugorac NJ (2008). Interferon-lambda1 (interleukin-29) preferentially down-regulates interleukin-13 over other T helper type 2 cytokine responses in vitro.. Immunology.

[13] Dai J, Megjugorac NJ, Gallagher GE, Yu RY, Gallagher G (2009). IFN-lambda1 (IL-29) inhibits GATA3 expression and suppresses Th2 responses in human naive and memory T cells.. Blood.

[14] Kotenko SV (2011). IFN-lambdas.. Curr Opin Immunol.

[15] Nordström I, Rudin A, Adlerberth I, Wold A, Saalman R (2010). Infection of infants with human herpesvirus type 6 may be associated with reduced allergic sensitization and Th2 development.. Clin Exp Allergy.

[16] Svensson A, Almqvist N, Nordström I, George-Chandy A, Eriksson K (2010). Exposure to Human Herpes Virus type 6 protects against allergic asthma in mice.. Journal of Allergy & Therapy.

[17] Osterlund PI, Pietila TE, Veckman V, Kotenko SV, Julkunen I (2007). IFN regulatory factor family members differentially regulate the expression of type III IFN (IFN-lambda) genes.. J Immunol.

[18] Izaguirre A, Barnes BJ, Amrute S, Yeow WS, Megjugorac N (2003). Comparative analysis of IRF and IFN-alpha expression in human plasmacytoid and monocyte-derived dendritic cells.. J Leukoc Biol.

[19] Yu HR, Huang HC, Kuo HC, Sheen JM, Ou CY (2011). IFN-alpha production by human mononuclear cells infected with varicella-zoster virus through TLR9-dependent and -independent pathways.. Cell Mol Immunol.

[20] Lund J, Sato A, Akira S, Medzhitov R, Iwasaki A (2003). Toll-like receptor 9-mediated recognition of Herpes simplex virus-2 by plasmacytoid dendritic cells.. J Exp Med.

[21] Fiola S, Gosselin D, Takada K, Gosselin J (2010). TLR9 contributes to the recognition of EBV by primary monocytes and plasmacytoid dendritic cells.. J Immunol.

[22] Svensson A, Nordström I, Rudin A, Bergström T, Eriksson K (2011). Enveloped virus but not bacteria block IL-13 responses in human cord blood T-cells in vitro..

[23] Lin SC, Kuo CC, Tsao JT, Lin LJ (2012). Profiling the expression of interleukin (IL)-28 and IL-28 receptor alpha in systemic lupus erythematosus patients.. Eur J Clin Invest.

[24] Arasteh J, Ebtekar M, Pourpak Z, Pourfatollah AA, Hassan ZM (2010). The effect of IL-28A on human cord blood CD4+ T cells.. Immunopharmacol Immunotoxicol.

[25] Liu BS, Janssen HL, Boonstra A (2011). IL-29 and IFNalpha differ in their ability to modulate IL-12 production by TLR-activated human macrophages and exhibit differential regulation of the IFNgamma receptor expression.. Blood.

[26] Megjugorac NJ, Gallagher GE, Gallagher G (2009). Modulation of human plasmacytoid DC function by IFN-lambda1 (IL-29).. J Leukoc Biol.

[27] Jordan WJ, Eskdale J, Boniotto M, Rodia M, Kellner D (2007). Modulation of the human cytokine response by interferon lambda-1 (IFN-lambda1/IL-29).. Genes Immun.

[28] Koltsida O, Hausding M, Stavropoulos A, Koch S, Tzelepis G (2011). IL-28A (IFN-lambda2) modulates lung DC function to promote Th1 immune skewing and suppress allergic airway disease.. EMBO Mol Med.

[29] Peter M, Bode K, Lipford GB, Eberle F, Heeg K (2008). Characterization of suppressive oligodeoxynucleotides that inhibit Toll-like receptor-9-mediated activation of innate immunity.. Immunology.

